# Correlations between Microbiota Bioactivity and Bioavailability of Functional Compounds: A Mini-Review

**DOI:** 10.3390/biomedicines8020039

**Published:** 2020-02-20

**Authors:** Emanuel Vamanu, Florentina Gatea

**Affiliations:** 1 Faculty of Biotechnology, University of Agronomic Science and Veterinary Medicine, 59 Marasti blvd, 1 district, 011464 Bucharest, Romania; 2 Centre of Bioanalysis, National Institute for Biological Sciences, 296 Spl. Independentei, 060031 Bucharest, Romania; florentina.gatea@incdsb.ro

**Keywords:** probiotic, bioaccesibility, polyphenols, absorption, pattern

## Abstract

Numerous studies have demonstrated the role of the microbiota in supporting the physiological functions, owing to its metabolomic component. The presence of biocomponents generally leads to the correction of the microbial pattern correlated with the reduction of oxidative pressure. This study aims to present the main processes that correlate the bioavailability and bioactivity of some functional components through the action of the human microbiota. The use of probiotics and prebiotics is an innovative manner involving alternatives that increase the bioavailability of certain natural or metabolic components has been proposed. Probiotic strains (*Saccharomyces cerevisiae* or *Lactobacillus (L.) plantarum*) may represent an intermediary for increasing the antioxidant bioactivity, and they may be administered in the form of a biomass enriched with functional compounds, such as phenolic acids. The limiting effect of gastrointestinal transit is, in several cases, the key to the biopharmaceutical value of new products (or supplements). The identification of newer ways of formulating supplements also involves the compatibility of different types of products, the testing of bioaccessibility, and the elimination of biotransformations.

## 1. Introduction

Numerous studies on gut microbiota have confirmed its role in the appearance and evolution of some diseases like inflammatory bowel disease, obesity, diabetes, cardiovascular diseases, cancer, allergy, and neurological disorders [1]. Various factors are known to influence the microbiota of an individual, including diet, genetic factors, and the administration of certain drugs (e.g., antibiotics). These changes may persist for several days, weeks, or even permanently affect the microbiota, inducing dysbiosis. The health status of the microbiota directly influences the assimilation of some nutrients, compounds from food or compounds administered in order to maintain the health such as dietary supplements based on natural compounds, as well as their metabolism and bioavailability. Some of these compounds, which are derived from exogenous sources (e.g., polyphenols) may improve the status of the microbiota and may reduce its oxidative stress [2]. Defined as non-nutrients, secondary plant metabolites, antioxidants, or bioactive substances in plants [3], polyphenols represent a class of compounds that has been studied not only because of their biological properties (such as immunomodulatory and anti-inflammatory [4,5,6], cardioprotective [7,8], anti-diabetic and anti-obesity [9,10], anti-cancer [11,12,13], neuroprotective [14,15], anti-asthmatic [16,17], anti-hypertensive [18], anti-aging [8], hepatoprotective [19,20], antibacterial [21,22], anti-fungal, and antiviral [23,24]), but also because they are present in most types of diet. The main sources of polyphenols in the diet include fruits, vegetables, tea, and coffee, but they can also come from the administration of various food supplements.

The data on the role of the human microbiota in sustaining the general state of health are increasingly being used in the scientific community. The importance of the microbial pattern is determined by its involvement in the metabolism of different bioactive compounds resulting from the consumption of food and/or the administration of functional supplements. Together with probiotics and prebiotics, natural biocomponents (such as polyphenolic acids) are a target in the fight against chronic pathologies. These targets are determined by inflammatory progression, which is the true cause of most degenerative pathologies, and sustained by the constant pressure of oxidative stress [25].

The metabolism and process of absorption of polyphenols has been intensively studied considering their high therapeutic potential and the involved benefits to human health, also well known for some of polyphenols [26]. On ingestion, a part of them gets absorbed at the level of the small intestine, and most of the polyphenols are transformed by the microbiota in the colon. Seemingly, only a part of the bacteria strains can metabolize these compounds, and compositional changes of the bacterial strains or dysbiosis can affect the process. The use of dietary supplements containing beneficial bacteria, which are capable of modulating the microbiota for transforming these substrates, is an alternative with significant health implications. However, in the colon, the role of polyphenols is not only to act as a substrate for the enzyme apparatus of the microbiota or as a carbon source, in fact, they can also modulate the population of microorganisms due to the antimicrobial effect that many polyphenols have [27].

The antimicrobial effect is one of the most important properties from which the interest for these compounds starts. The antimicrobial effects of polyphenols are exerted by several mechanisms, such as: direct action on certain target bacterial strains, reducing the adhesion capacity of pathogenic strains, or disrupting ionic fluxes at the cell membrane [22,28]. One of the main mechanisms by which these compounds act is by stimulating the multiplication of favorable strains [29] for correcting the microbial and metabolomic patterns [1]. The bioavailability of the functional components represents the property that controls the bioactivity in vivo. For example, gallic acid and its derivatives are directly involved in the synthesis of short-chain fatty acids (SCFAs). Their biological action is mediated through the human microbiota, leading to an improvement of the physiological functions [30]. Thus, the clinical yield is controlled in such situations by the percent of bioavailability. In general, it is considered to be low [31]. In several cases (such as in the administration of functional products), the control of the clinical efficiency occurs through the products that result from the degradation of the majority compound, that determines the known general effect [32]. These data are usually considered based on the knowledge gained from traditional medicines.

## 2. Gut Microbiota and Polyphenols

The interaction between the polyphenols and the microbiota can be approached both from the perspective of how they are metabolized by the microbiota and also how they can modulate the microbiota to increase their impact in the prevention and improvement of some diseases [27,33].

Although in vivo studies provide the most valuable data about these interactions, in vitro studies involving the reproduction of gastrointestinal conditions provide extremely useful information for understanding the process of these interactions, which are not yet fully elucidated for their use in practice; for example, in obtaining a new type of dietary supplements.

Due to the chemical diversity of polyphenolic compounds, it is difficult to predict how the microbiota can respond to their action. They differently modulate the microbiota, favoring the development of some bacterial strains, and act like a prebiotic. A recent study revealed that the *Curcuma longa* extract with a high curcumin content (Table 1) modulates the microbiota of patients with hypertension by improving the ratio of SCFAs, which strongly influences the *Enterobacteriaceae* group and negatively influences the *Bacteroides-Prevotella-Porphyromonas* groups [29]. Polyphenols from the *Lonicera caerulea L. berry* reduced the *Firmicutes*/*Bacteroidetes* ratio in the fecal microbiota of a mouse model with an induced high-fat diet (HFD) and increased the relative abundance of *Bacteroides* and *Parabacteroides*. The results suggested the potential use of these extracts in microbiota modulation for the attenuation of inflammation present in non-alcoholic fatty liver disease [34]. The administration of a Concord grape extract stabilized with soy protein into mice on HFD showed a significant increase in *Akkermansia muciniphila* in parallel with the corresponding decrease in *Firmicutes* to *Bacteroidetes*. Grape extract modulates the gut microbiota and decreases the intestinal and systemic inflammation by improving the metabolic parameters [35].

Determination of the flavanols (-) epicatechin and (+) -catechin monomers in a batch-culture fermentation system revealed that they are metabolized to 5- (3′,4′-dihydroxyphenyl) -γ-valerolactone, 5-phenyl-γ-valerolactone, and phenylpropionic acid via a transformation, that, for (+) catechin, first involves a conversion to (+) epicatechin. As a result, catechin significantly increased the growth of the *Clostridium coccoides*–*Eubacterium rectale* group, *Bifidobacterium* spp. and *Escherichia coli*, which, in total, had a significant inhibitory effect on the growth of the *C. histolyticum* group. The influence of epicatechin was less significant, leading to a more pronounced increase of the *C. coccoides–E. rectale* group [39]. The same monomeric flavanols that are present in a more complex matrix containing their oligomeric derivatives—cocoa drinks—were tested on human volunteers, as a daily dose (Table 1), resulting in an increase in the *L.* and *Enterococcus* spp. populations and a decrease in the *C. histolyticum* group. These results suggest that the two flavanols, which are present in a more complex matrix that brings an extra source of energy, can have far more beneficial effects on the microbiota [40].

After testing the individual effect of some polyphenols (such as naringenin, naringin, hesperetin, hesperidin, quercetin, rutin, and catechin) on the growth of bacterial strains representative of the human intestinal microbiota (such as *Bacteroides galacturonicus* and *L.* sp. *Escherichia coli*) revealed that their inhibition or activation seems to be in the function of the polyphenol structure. Catechin and rutin have no impact on bacterial strains. Aglycons of naringenin and hesperidin inhibit all bacteria. Quercetin had a strong impact, followed by naringenin and hesperidin [46]. Therefore, the manner in which polyphenols influence the microbiota, in general, and how each type of bacterial population in particular may be related to their structure, the matrix they are a part of, the dose of administration, and the type of diet, is a much more complex phenomenon. The administration of resveratrol—a low bioavailability polyphenol—along with a HFD to C57BL/6 mice has been shown to be beneficial in a dose-dependent manner for maintaining the microbiota’s compositional stability. The results showed that the administration of resveratrol does not reverse the changes induced by the HFD, but leads to a significant increase in *Deferribacteraceae* and a decrease in *Desulfovibrionaceae* [41].

## 3. Probiotics Strains and Bioavailability of Functional Compounds

The administration of probiotics, especially the strains of lactic bacteria, is currently an accepted method of controlling the microbial pattern at the microbiota level [47]. Although this is not a long-term solution, it can correct the possible temporary excesses of some drugs that intervene in the establishment of colonic dysbiosis [48]. The plasticity of the microbiota offers a high degree of acceptability of these strains, which eliminates the possible negative effects that may be caused by the introduction of new strains [49]. In the short-term, a correction can occur, but the high rate of rejection of the strains after cessation of administration is due to molecular incompatibility [50].

Thus, increasing the biopharmaceutical importance can be achieved in an innovative manner by enriching the biomass, especially yeasts (*Saccharomyces* sp.) with functional compounds such as polyphenolcarboxylics [51]. Normally, they are used as a carbon source by probiotic strains of the genus *Lactobacillus* and *Bifidobacterium*, but their role is much more complex. Their catabolism regulates the synthesis of SCFAs and corrects the microbial pattern in degenerative pathologies [2,29]. Partial in vitro studies conducted at the Faculty of Biotechnologies, Bucharest, Romania showed that the polyphenols present in matcha tea and green tea adopt a selective metabolization, the assimilation rate of which is about 20% (data unpublished as of yet). An increase, depending on the substrate, of the antioxidant status of the biomass was observed, with the effect of amplifying the bioactivity in vitro after their valorization.

It can thus be assumed, at least in vitro, that it has the capacity to adapt to stress as the exposure time increases, along with an intensification of the fermentative action. The claim is justified by increasing the amount of CO_2_ formed, which can support the use of the carbon source (data unpublished as of yet), by probiotic yeasts (*S. cerevisiae* and *S. boulardii*). This aspect has been demonstrated by previous studies that have shown the selective use, by certain strains, of bioactive components in extracts [29,52]. Increasing the number of favorable strains in the microbial pattern of the microbiota can thus have two causes, as detailed below:

- The direct increase in the number of cells—a phenomenon that is much more difficult to prove—due to the lack of any direct scientific evidence;
- Reduction (inhibition) of some pathogen strains and the release of ecological niches where *Lactobacillus* strains, for example, can proliferate [29].

On the other hand, the modulation of the microbial pattern does not have any proof of the persistence over time of the positive effect [53]. It is therefore assumed that the administered probiotics show this limiting effect. Thus, it can be considered that the limitation could be the result of the depletion of the carbon source that supports the rapid rate of multiplication. In contrast, if the effect is retained, it could be assumed that the elimination of a limiting factor (oxidative stress, [52]) may represent a new direction of valorization (research) that involves a multidisciplinary study [54].

### Effect of Probiotics Strains on Bioavailability of Functional Compounds

In the recent years, the use of probiotics for microbiota modeling has proven to be an extremely promising alternative with beneficial health effects. To date, their contribution has been proven to enhance the nutrients’ bioavailability and reduce the risk of developing diseases. In cardiovascular diseases, a possible mechanism of action of probiotics is via the inhibition of hepatic lipogenesis and the lowering of blood glucose and insulinemia [55].

Increasing the synthesis of small-chain fatty acids due to the presence of probiotics seems to be a possible way of influencing the colon homeostasis by activating the free fatty acid receptors involved in regulating the immune system and secreting glucagon-like peptides-1 (GLP-1), which stimulates insulin secretion in pancreatic cells. In this way, autoimmune diseases such as type 1 diabetes can be managed [56].

The study of the microbiota for the promotion of functional food or dietary supplements is gaining new dimensions in the context of new research. The probiotic *L. paracasei* A221 (A221), for example, has proven to have a major influence on the functionality and bioavailability of kaempferol-3-o-sophroside (KP3S; a kaempferol-glucoside contained in kale). A221 strain can convert KP3S into aglycone by its unique beta-glucosidase activity; therefore, its administration plays an important role in the bioavailability of kaempferol and not least in increasing the anti-aging activity of KP3S in vivo [57]. The impact on the microbiota of a polyherbal formulation (TFLA) composed of equal quantities of *Emblica officinalis*, *Terminalia chebula*, and *Terminalia belerica* rich in phenolic, acids, flavonoids, and condensed and hydrolyzable tannins has increased considerably when it is administered with a probiotic consisting of *L. plantarum*, *L. fermentum*, and *Bifidobacterium infantis*. A polyphenolic prebiotic and probiotic formulation manifested synergistic effects, leading to an increase in the ratio of *Bacteriodetes* to *Firmicutes*, *Lactobacillus* spp., *Ruminococcus* spp. concentration and a decrease for *Enterococcus*, *Staphylococcus*, and *E. coli* [58].

A new approach to the probiotic–polyphenols relationship is the use of polyphenols to improve the physiological functionality of probiotics. This approach is extremely interesting in the context of developing new pharmaceutical and food probiotic and prebiotic formulas. The physiological functionalities of *Lactobacillus* strains with a probiotic potential have been tested at different concentrations of quercetin and resveratrol. The results show that the physiological functionality of bacterial strains can be enhanced in the presence of polyphenols. These improvements depend on the type and concentration of polyphenol and *Lactobacillus* strain tested. Quercetin showed better protective effects than resveratrol and among the tested strains, the best results were obtained for *L*. *fermentum* and *L. plantarum* strains [59].

## 4. Predictive Microbiota Response

The bioavailability of bioactive compounds is a predictive model of the effect that a functional product can determine. At present, different methods are used to improve the bioavailability and to increase the microbiota’s metabolic activity [60]. The whole process, which correlates with the physiological state of the target group, depends on the bioactive molecule and can influence the fermentative state if it has a concentration that determines a response in the microbial pattern [61].

The restoration of eubiosis has proven to be the key factor in clarifying the role of microbiota in the development of certain autoimmune pathologies. According to this study, the interaction of vital functions with the microbial pattern is the cause that leads to the appearance of pathologies that have no clear resolution in the medical field [48]. New types of drugs/supplements, with a clear direction toward pharmaceutical probiotics and prebiotic formulas, are seen as the solution to the physiological impairments that have arisen due to dysbiosis [62]. Increasing the number of strains is an alternative to the well known probiotics based on the species of the genera *Lactobacillus*, *Bifidobacterium,* and/or *Streptococcus* [48], although only the introduction of new strains cannot guarantee microbiota modulation over a period of time. The multitude of limiting factors (from genetic to behavioral) makes the success of such strategies a small one, in the current context [63].

The mechanisms that consider the bioavailability of some phytochemicals in the improvement of the metabolic syndrome are not well known. The biopharmaceutical effect depends on their chemical structure and their individual bioassimilation ability. A significant limiting factor is the lack of standardization, and the administration period is one of the two essential causes of reducing the efficacy in vivo [64].

An important example is the use of mushrooms with pharmaceutical potential (Table 1). This substrate is useful in combating cancer progression because the action of the bioactive molecules they contain expresses a low toxicity, with good tolerance in the human body [65]. Products that use components from wild mushrooms, and not only medicinal species, determine the restoration of eubiosis to target the population groups. By balancing the microbial pattern, it is possible to promote the synthesis of compounds that act as biomarkers towards improving health status. Such results can be considered to directly correlate with predictable metabolomic activity [48,52].

In fact, the diversity of molecules present in a functional compound (extract) cannot provide a predictable metabolomic pattern. This is an essential gap that could influence product bioactivity. This aspect may be significant in the consideration of nutraceuticals being clinically important for people suffering from diseases associated with oxidative stress [66]. The analysis of the metabolic differences determines the optimization of the potential biomarkers associated with the administration of compounds with a functional role. Thus, the studies characterizing the microbiota and the metabolomics pattern have led, through in vitro studies, to the better understanding of the physiological response, which should correlate with studies demonstrating bioavailability as an active support for the expression of bioactivity in vivo [67]. Metabolites produced by the microbiota play a significant role in modulating the physiological functions due to bioactivity. The new microbiota-generated compounds act as bioactivators [68].

## 5. The Causes of Disruption of the Microbiota-Mediated Response

Dysbiosis is the primary cause of the decreased efficiency of physiological response mediated by microbiota bioactivity. Changing the microbial balance is an effect that arises from different causes that can often act synergistically. If the temporary balancing by probiotics has a positive effect, over a period of time, this effect will be one of diminishing bioassimilation of molecules of pharmaceutical importance (e.g., antibiotics, flavonoids) [69]. The use of the main natural bioactive components as a carbon source [29] is a rarely accepted solution, but it does explain how the microbiota responds to the administration of some compounds that are considered to possess antibacterial effects.

Individual variability at the microbiota level is the key factor influencing the bioavailability of compounds in functional products [70]. Biotransformation of ingested components conditions the bioactivity, through the microbiota’s metabolizing capacity. Biotransformation is a process that is similar to the recognition process between substrate and receptor [71]. The process is conditioned by the classes of ingested compounds and the basic chemical structure. The bioavailability process will be influenced by the relationship between biotransformation and catabolization due to the chemical structure. The individual variation will balance the whole process, which indirectly controls the bioactivity. This equilibrium will determine which molecules may be active for certain target population groups, which increases the resultant biopharmaceutical value of the functional products [72].

The disruption of microbial eubiosis (the critical point for establishing dysbiosis) is the cause of physiological impairments associated with microbial infection. The process is correlated with a decrease in the number of strains of the genera *Lactobacillus, Bifidobacterium*, and *Firmicutes* and with an increase in pathogens, mainly *E. coli* [73]. This process is often associated with urinary tract infections, poor bioavailability of nutritional compounds and the disturbance of the ratio of SCFAs [2,29].

Although probiotics are considered as products with guaranteed effect, which increases with the amount ingested, new theories on in vivo bioactivity contradict the known data [49,74]. The eubiosis considered to be established is a temporary one, in fact, molecular interactions lead to a microbiota imbalance and a different response over time. This does not mean that people will immediately experience a negative physiological effect, but that it would be a change that occurs over time as the microbial pattern is reshaped. Such an interpretation will develop new theories on functional supplements that are considered to have a known effect. The remodeling of the microbial pattern extends the research performed so far and opens up theories that will reconsider the introduction of new strains into the human microbial ecosystem by modifying/decreasing the bioavailability of some essential functional compounds [75]. This is a secondary explanation for the reduction of colonic bioavailability, microbial plasticity, and the incidence of degenerative diseases [76].

The overdose of pharmaceuticals (such as antibiotics) limits the plasticity of the metabolomic pattern in order to maintain a favorable health status. A much more useful solution for the long term is the use or administration of the fibers that lead to the synthesis of butyrate, while simulatenously preserving the integrity of the microbial pattern. Such products support the individual eubiotic state [33]. In this situation, no new molecular relationships is expected to emerge, and the intrinsic effects of this relationship can only be quantified in the long term [77].

The intestinal ecosystem has been shown to be responsible for xenobiotic biotransformation, and several recent studies have proven it to be an essential factor in the pharmacokinetics of orally administered drugs. This aspect has direct implications for bioavailability after oral administration [78]. The metabolism process of xenobiotics is an enzymatically controlled one and depends on the age of the individual. Enzyme synthesis varies with diet, and it also depends on the spatial distribution of the genera that compose the key pattern of the target-group microbiota [2,79]. From a clinical perspective, an increase in the number of enzymes with age has been noted, and a prolonged exposure to a compound implies a modulation of the metabolism capacity, with a direct effect on the bioavailability of drugs [80]. Such studies can explain the different roles that the microbiota play in the bioavailability of drugs in comparison to other functional components.

The absorption of different types of biomolecules depends on the molecular mass and their chemical structure. Polyphenolic compounds have been recognized to have a low bioavailability, and their effect is often mediated by intermediates [29]. A recent study assimilates these components after digestion in the upper segments as xenobiotics [81]. They are assimilated only after the enzymatic action of the microbial community in the colon and do not have any direct critical effect. If other xenobiotics cause a direct alteration of the microbial pattern, they have a different function [82], such as a modulatory one, with the clinical effect exerted by the expressed bioactivity [2].

## 6. Dietary Fiber and Their Role in the Bioavailability of Phenolic Compounds

The bioavailability of polyphenols in the gastrointestinal tract, as well as their catabolism at the microbiota level, may be influenced by the presence of fibers. Simultaneously, the presence of fibers stimulates the microbiota, as they are the main source for SCFAs synthesis. This type of interaction is not fully understood yet [83]. A recent study showed that the presence of highly fermented fibers can inhibit phenolic acid production from the catabolization of rutin by human fecal bacteria. In vitro experiments using the same highly fermented fibers showed that the presence of rutin or quercetin in the medium did not affect SCFAs synthesis [84]. A long-term diet with water-soluble dietary fibers (such as pectin, soybean fiber, and guar gum) and quercetin-3-O-glucosides mixture supplementation to rats led to increased SCFAs production and the improved bioavailability of quercetin glycosides. The increased levels of quercetin in the blood and urine for rats supplemented with soybean fiber and quercetin glycosides may be attributed to the use of fiber as a carbon source and the suppression of quercetin degradation by the microbiota [85].

Polyphenols interact with all types of fibers, and the nature of the interactions is defined by the chemical structure of that fiber and by the environmental conditions. These interactions may be hydrophobic, hydrogen bond, van der Waals, non-covalent, non-ionic, or weak electrostatic interaction [86]. As a result of these interactions, some polyphenols become less bioavailable in the small intestine and more in the large intestine. Therefore, fibers play an important role in the bioavailability of polyphenols in the colon, and polyphenol-binding fibers brings health benefits [87].

Mulberry leaf extracts are commonly used as a natural remedy in type 2 diabetes. Its efficacy in lowering the blood glucose levels has been demonstrated by numerous studies [88]. A recent study showed that the presence of fibers increased the efficiency of polyphenolic extract from mulberry leaf, and the polyphenol–fiber mixture influences the gut microbiota (Table 1). Their synergistic effect resulted in the reduction of *Firmicutes* abundance, the downstream of *Clostridiales* and *Lachnospiraceae*, and a high concentration of butyrate [89]. The effects of synergic polyphenols and dietary fibers has also been described by a study on gastrointestinal digestion of two grape pomace extracts (GPEs) [90]. The in vitro colonic digestion of GPEs administered in a single dose or continuously (14 days) resulted in the significant increase of SCFAs and ammonium ions production, as well as an increase in the count of *Lactobacillus* and *Bacteroides* groups. The presence of phenolic metabolites in the colon during the frequent administration of GPES indicate the bioavailability of polyphenols and has been assigned to the main GPE components, dietary fibers, and polyphenols.

## 7. Perspectives in Approaching Studies on Microbiota and Bioavailability of Functional Compounds

**Figure 1:**
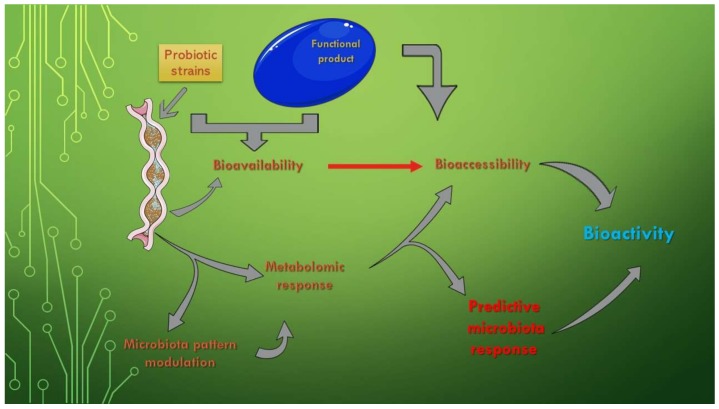
The effects of microbiota bioactivity and bioavailability of functional compounds. This Figure was obtained in part by using images from Servier Medical Art, licensed under CC-BY 3.0.CC BY 3.0, and the PowerPoint program from the Microsoft Office 2016 software package (Microsoft Corporation, Redmond, WA, USA).

The existing data on the composition, properties, and role of the microbiota recommend the use of bacterial profiles as predictors of disease, as manipulating the gut microbiota is a promising alternative for the treatment of diseases. The question that arises in this case is whether the long-term use of functional foods is sufficient to restore the health of the microbiota or whether other interventions, such as fecal microbiota transplantation or genetic manipulation of the microbiota, are required to complete their actions [91] (Figure 1).

The bioavailability of functional compounds can be used for enhancing the effects of one of the two clinical forms (Crohn’s Disease—CD and Ulcerative Colitis—UC) of Inflammatory Bowel Disease (IBD). This is a significant aspect because IBD is sustained by a chronic inflammatory process, which directly leads to disruption of the microbiota pattern [92]. The independent use of probiotics only temporarily ameliorates clinical manifestations because they do not intervene in the active reduction of the inflammatory process. The assimilation of functional components, especially of target compounds, is one of the complementary evolutionary solutions in this regard. These new product types, biomass-enriched with certain functional components, are addressed to clinical forms that have not shown a clear improvement after single biomass administration, such as UC [93]. Figure 1 also represents a schematic form of the physiological role that the increase of the bioavailability of some products (of the bioaccessibility of all the components of its composition) has on the modulation of the metabolic response. In addition, by knowing the effect of the entire functional fingerprint, a prediction of the physiological effect and a determination of the bioactivity after administration can be made.

Modulation of the metabolomic pattern for SCFA could represent a breakthrough in IBD researches. Butyric acid is the target of this response because the ratio of the three main acids varies with the target groups and decreases with the passage from one segment to another. The role of these acids in controlling inflammatory proliferation increases not only with the number of favorable strains, but also with the decrease of oxidative stress [29,94]. The prebiotic role of nutraceuticals is essential in the proliferation of strains synthesizing butyrate (microbial modulation), like food fibers. This aspect has a major influence in reducing the chronic inflammation that favors the appearance of tumors, a process relevant especially for the terminal segment of the human colon [94].

The use of favorable strains (as probiotic products) offers a new perspective through its association with certain molecules with a functional role, and directly influences the bioavailability process. Future research will need to evaluate the role that newly introduced strains can play in the microbiota–bioavailability relationship. Diversity and spatial spread play an important role in determining the optimal action in the interaction with the functional compound in the human colon. This process evolves gradually with administration, and results in an increase in the immune response [95]. The restoration of eubiosis, with increasing bioavailability, is partially associated with the administration of functional compounds [73,96], unless they are degraded by the interaction with the physico-chemical limiting factors of the gastrointestinal tract. Reducing the action of limiting factors that influence bioavailability is the main objective of research on the effect of functional compounds (such as phenolic compounds), by mitigating possible xenobiotic effects.

The prevention of oxidative stress through the consumption of phenolic compounds is a key solution for reducing the chronic pathologies associated with the establishment of dysbiosis. Although they are the main functional components in herbal products, phenolic compounds possess several characteristics that are not fully known. Bioaccessibility and biotransformation of phenolic compounds manifest, at the microbiota, a key point that affects their bioactivity and on which the physiological response depends [97].

Functional products are widely available in the present market. Research on improving the health status by administering these products should be undertaken by the biopharmaceutical industry toward developing newer products with the optimum efficiency in vivo. These should not be based solely on the combination of compounds with a known biological effect from the traditional phytomedicines [98].

## 8. Conclusions

The new evidences in microbiota research confirm the role played by nutrients in modulating it. The restoration of the gut microbiota commonly associated with the occurrence of diseases by increasing the bioavailability of some natural compounds is much easier than using pharmaceutical alternatives. We believe that studies on the interaction of microbiota with polyphenolic compounds and other factors may influence the restoration process and human well-being.

## Figures and Tables

**Table 1:** The interaction between the microbiota and polyphenols.

Polyphenols	Related Bacteria	Mechanisms	Effects	References
Curcumin	*Enterobacteriaceae* group phylum Firmicutes	Antimicrobial action	Increase antioxidant status/ Microbiota pattern modulation	[28]
Hesperetin/ Naringenin	*Helicobacter pylori,* *Escherichia coli*, *Salmonella aureus*	Antimicrobial action	Upper part of digestive tract Modulate gut microbiota fingerprint	[36]
Vanillic acid and Gallic acid	Microbial diversity was altered	Changes in the bioavailability of phenolic acids	Induced dysbiosis	[37]
Catechin	*Eubacterium* sp.	Biotransformed by gut microbiota	Excretion in the feces, Xenobiotic	[38]
Epicatechin	*Bacteroides*, *Firmicutes*, *Bacteroidetes*	Decrease serum levels of inflammatory cytokines including IL-2, IL-6, TNF-α, and MCP-1	Bacterial ratio modulation	[34]
Concord grape polyphenols	*Akkermansia muciniphila* *Firmicutes* *Bacteroidetes*	A lower intestinal expression of inflammatory markers (TNFα, IL-6, inducible nitric oxide synthase) and a gene for glucose absorption (Glut2)	Modify the structure of the gut microbiota	[35]
Epicatechin Catechin	*Clostridium coccoides–Eubacterium rectale,Bifidobacterium* spp. and *Escherichia coli*	Formation of 5- (3 ′, 4′-dihydroxyphenyl) -γ-valerolactone, 5-phenyl-γ-valerolactone and phenylpropionic acid	Bacterial ratio modulation	[39]
Cocoa flavanol	*Enterococcus* spp. *C. histolyticum*	Changes in C-reactive protein serum concentrations	Bacterial ratio modulation	[40]
Resveratrol	*Coriobacteriaceae* and *Desulfovibrionaceae*	Decreased serum interleukin-1 and tumor necrosis factor-alpha,	Prevents chronic inflammation, decreased oxidative stress, bacterial ratio modulation	[41]
Stilbenes	*Bifidobacterium, Lactobacillus* and *Akkermansia*	Alleviate symptoms of gut inflammation	prebiotic-like effect, modulation of pro-inflammatory cytokines	[42,43]
Caffeic acid, chlorogenic acid, ferulic acid, coumaric acid	*Bifidobacterium, Lactobacillus*	Microbiota pattern modulation	Increase butyrate production	[44,45]
